# Preparación para la muerte de personas mayores: perspectivas de equipos de salud primaria, familiares y cuidadoras principales

**DOI:** 10.1590/0102-311XES171023

**Published:** 2024-11-22

**Authors:** Jame Rebolledo-Sanhueza, Verónica Aliaga-Castillo, María Luz Bascuñán-Rodríguez, Julia Acuña-Rojas, Kurt Kirsten-Toro, Rocío Briceño-González, María Constanza Cornejo-Guerrero, Gabriela Huepe-Ortega

**Affiliations:** 1 Facultad de Medicina, Universidad de Chile, Santiago, Chile.; 2 Hospital Santiago Oriente Dr. Luis Tisné Brousse, Santiago, Chile.; 3 Centro Comunitario de Salud Mental de Macul, Macul, Chile.

**Keywords:** Cuidados Paliativos, Actitud Frente a la Muerte, Muerte, Anciano, Palliative Care, Attitude to Death, Death, Aged, Cuidados Paliativos, Atitute Frente a Morte, Morte, Idoso

## Abstract

La preparación para la muerte debe ser parte integral de los cuidados de fin de vida y una preocupación para la salud pública. El objetivo de este estudio fue describir los significados y prácticas de la preparación para la muerte de personas mayores en fin de vida desde las perspectivas de equipos de salud primaria, familiares y cuidadoras principales. Se realizó un estudio cualitativo, por medio de grupos focales y entrevistas individuales, con una muestra de 99 personas, 45 profesionales y técnicos de atención primaria de salud, 30 cuidadoras(es) y 16 familiares. Los resultados se organizaron en tres dimensiones: (1) significados sobre la muerte e implicaciones para la preparación para la muerte, destacando el dolor de la muerte, el alivio del sufrimiento y la mediación de las creencias religiosas; (2) prácticas de preparación para la muerte de las personas mayores, incluyendo la dificultad para hablar sobre la muerte, la búsqueda de la tranquilidad y las preferencias fúnebres como medios para la preparación; y (3) prácticas de los equipos de acompañamiento a las cuidadoras y familias, que incluyen el apoyo psicológico y espiritual durante el cuidado y la posibilidad de cerrar los ciclos de asistencia domiciliaria durante el duelo. Se discute la controversia de la preparación para la muerte para los participantes del estudio y los desafíos de la atención primaria para incluirla dentro de los cuidados paliativos. En conclusión, este estudio muestra la importancia de incluir estrategias de preparación para la muerte en el ámbito de los cuidados de fin de vida en la atención primaria, para promover el buen morir como parte integral del buen vivir.

## Abstract (en)

Preparing for death should be an integral part of end-of-life care and a public health concern. The objective of this study was to describe the meanings and practices of preparing for the death of older adults at the end of life from the perspectives of primary health care teams, family members and primary caregivers. A qualitative study was carried out through focus groups and individual interviews with a sample of 99 individuals, 45 primary health care professionals and technicians, 30 caregivers and 16 family members. The results were organized in three dimensions: (1) meanings about death and implications for preparing for death, highlighting the pain of death, suffering relief, and mediation of religious beliefs; (2) the practices of preparation for death of older adults, including the difficulty of talking about death, the search for tranquility and funeral preferences as a form of preparation; and (3) practices of teams that accompany caregivers and families, including psychological and spiritual support during care and the possibility of closing home care cycles during grief. The controversy of preparing for death for study participants and the challenges of primary care to include it in palliative care are discussed. In conclusion, this study shows the importance of including preparation for death strategies in the end-of-life care setting in primary care to promote a good death as an integral part of a good life.

## Abstract (pt)

A preparação para a morte deve ser parte integrante dos cuidados no fim da vida e uma preocupação de saúde pública. O objetivo deste estudo foi descrever os significados e as práticas de preparação para a morte de pessoas idosas no final da vida a partir das perspectivas das equipes de atenção primária à saúde, dos familiares e dos cuidadores primários. Foi realizado um estudo qualitativo por meio de grupos focais e entrevistas individuais com uma amostra de 99 pessoas, 45 profissionais e técnicos da atenção primária à saúde, 30 cuidadores e 16 familiares. Os resultados foram organizados em três dimensões: (1) significados sobre a morte e implicações para a preparação para a morte, destacando a dor da morte, o alívio do sofrimento e a mediação de crenças religiosas; (2) as práticas de preparação para a morte dos idosos, incluindo a dificuldade de falar sobre a morte, a busca de tranquilidade e as preferências funerárias como forma de preparação; e (3) práticas de equipes que acompanham cuidadores e famílias, incluindo apoio psicológico e espiritual durante o atendimento e a possibilidade de fechar ciclos de atendimento domiciliar durante o luto. A controvérsia da preparação para a morte para os participantes do estudo e os desafios da atenção primária para incluí-la nos cuidados paliativos são discutidos. Em conclusão, este estudo mostra a importância de incluir estratégias de preparação para a morte no cenário de cuidados no final da vida na atenção primária para promover uma boa morte como parte integrante de uma boa vida.

## Introducción

Si bien la muerte de personas mayores es un evento esperado, no está libre de complejidades en su abordaje como problema de salud pública. Considerando el envejecimiento poblacional, las peores condiciones de vida acumuladas y las limitadas capacidades de los sistemas sanitarios, cada vez más familias deberán enfrentar el proceso de muerte de una persona mayor en domicilio ^1^.

Mundialmente, entre el 2000 y 2019, las enfermedades crónicas no transmisibles han aumentado de un 60,8% a un 73,6% ^2^. Cada año causan 41 millones de muertes en todo el mundo ^3^. Las demencias han experimentado el mayor crecimiento, aumentando un 526% en las últimas dos décadas ^4^. En la Región de las Américas, más de 10 millones de personas viven con demencia y se estima que cada 20 años se duplicará este número, siendo América Latina y el Caribe los más afectados ^5^. En Chile, el 22,2% de las personas mayores de 60 años tiene algún grado de dependencia, con un predominio de mujeres y con diferencias significativas por ingresos económicos entre el primer (26,7%) y quinto quintil (14,2%) ^6^. Estos datos resaltan la necesidad de abordar la preparación para la muerte como un proceso central en los cuidados de fin de vida e indispensable para el buen morir ^7^.

Pese a no haber un concepto acabado sobre la preparación para la muerte, ha sido definida como los sentimientos de preparación y conciencia para la muerte inminente ^7^. También, como los ajustes psicológicos y prácticos para reconocer la muerte como un problema próximo ^8^, tomar conciencia de la muerte y dar permiso para morir ^9^.

La exigencia de los cuidados cotidianos en fin de vida, las trayectorias impredecibles de algunas enfermedades y las pérdidas del proceso de cuidado ^10^ producen en quienes cuidan mayor vulnerabilidad y exposición a estrés psicológico ^11^, lo que explicaría el no lograr sentirse preparado para la muerte de la persona cuidada ^12^. Al respecto, se reporta que uno a dos tercios de las personas cuidadoras no se sienten preparadas, a pesar de que el 85% lo considera de gran relevancia ^9^ ^,^ ^13^. También se ha observado que la menor preparación genera mayor riesgo de duelo complicado, depresión, trastorno de estrés postraumático y ansiedad ^7^ ^,^ ^14^.

No obstante, los esfuerzos han estado dirigidos en desarrollar avances biomédicos y tecnológicos que repercuten en una progresiva medicalización del morir y el silencio en torno al proceso de fin de vida ^15^. Estas prácticas convierten a la muerte en algo ajeno a nuestra cotidianeidad ^16^, por lo que es posible que existan diferencias culturales en las necesidades de las familias, lo que subraya la importancia de adaptar los cuidados en fin de vida a las prácticas, costumbres y creencias de ellas ^13^, junto a los desafíos propios de las inequidades económicas de las famílias ^17^ ^,^ ^18^. De esta forma, es plausible considerar que la diversidad de capitales económicos, culturales y sociales de las familias, influyen en las creencias y acciones en torno al cuidado de la persona y en la propia preparación para la muerte.

Se ha considerado que el cuidado en fin de vida, que incluye la atención paliativa, debe ser un servicio integrado y esencial de la atención primaria en salud (APS), tanto por razones éticas, de salud pública y financieras ^19^. La preparación para la muerte puede ser una herramienta pertinente en este nivel, dada la mayor accesibilidad del equipo y disponibilidad de tiempo favoreciendo la expresión emocional de pacientes y cuidadores ^12^ ^,^ ^20^. En Chile la APS está presente en todo el territorio nacional, cubriendo cerca del 80% de la población (afiliada al seguro público de salud), mayormente grupos de medianos y bajos ingresos económicos, quienes pueden recibir atención sin copago de parte de un equipo de salud interdisciplinario que trabaja bajo un enfoque territorial y familiar ^21^.

La preparación para la muerte ha sido señalada como un asunto prioritario para quienes cuidan ^8^ ^,^ ^22^, pero suele ser una necesidad omitida por los equipos sanitarios e, incluso, invisibilizada para el resto de la familia. Aunque las experiencias relacionadas con la muerte pueden parecer comunes, hay una falta de estudios suficientes en América Latina para respaldar a los profesionales de la salud. Faltan datos contextualizados en la APS desde la perspectiva de los actores involucrados y sobre las prácticas que afectan la preparación para la muerte. Por otro lado, en el contexto chileno, en que recientemente se promulgó la *Ley nº 21.375* ^23^ de octubre de 2021, que “*Consagra los cuidados paliativos y los derechos de las personas que padecen enfermedades terminales o graves*”, el abordaje de la preparación para la muerte en los cuidados de fin de vida por parte de los equipos de APS, conlleva desafíos y expectativas para su implementación que hace aún más relevante su estudio dado sus potenciales implicancias a nivel de salud pública.

En el marco de una investigación cualitativa más amplia, el objetivo de este trabajo es describir los significados y prácticas de la preparación para la muerte de personas mayores en fin de vida desde las perspectivas de equipos de salud primaria, familiares y cuidadoras principales.

## Metodología

Se utilizó un diseño cualitativo con alcance exploratorio, dado que el proceso de preparación para la muerte es aún inexplorado en nuestra realidad nacional ^24^ ^,^ ^25^.

La población fueron profesionales y técnicos de los equipos de APS que atienden a personas mayores en el final de su vida, así como cuidadoras(es) y familiares, de 8 comunas de la provincia de Santiago en la Región Metropolitana de Chile, con distintos niveles de desarrollo social comunal, según dimensiones de ingresos, educación y salud (Índice de Prioridad Social -IPS) ^26^.

Los criterios de inclusión fueron:

(a) Profesionales y técnicos de APS con experiencia de al menos un año en programas que asisten a personas mayores en fin de vida;

(b) Cuidadoras(es) que han vivido la muerte de la persona a su cuidado al menos un año antes y no más de tres;

(c) Familiares que hayan experimentado la muerte de su familiar con quien vivían al menos un año antes y no más de tres.

El criterio de exclusión para los tres casos fue tener un duelo complicado u otro problema afectivo en etapa aguda o sin tratamiento, autodeclarado.

Los tres tipos de participantes fueron convocados por un funcionario(a) de la dirección de salud de cada comuna, especialmente capacitado para ello.

La muestra estuvo constituida de 99 personas, 45 profesionales y técnicos de APS, 30 cuidadoras(es) y 16 familiares (Tabla 1). El grupo de familiares fue de complejo reclutamiento, con mayor negativa a participar aludiendo falta de interés y de tiempo.

El proceso de consentimiento informado se realizó al momento de invitar al estudio y al iniciar los grupos focales o entrevistas. Este proyecto fue aprobado por el Comité de Ética en Investigación en Seres Humanos de la Facultad de Medicina, Universidad de Chile (proceso nº 139, octubre de 2020).

**Tabla 1:** Descripción de los participantes.

Tipo de participante	n	Edad promedio (rango en años)	Género		Profesión o parentesco (n)
			Mujeres (n)	Hombres (n)	
Profesionales y técnicos equipo de APS	45	36 (23-61)	36	9	Técnicos en enfermería nivel superior (11), enfermeras/os (10), trabajadores/as sociales (6), kinesiólogos/as (5), psicólogos/as (5), médicos/as (4), nutricionistas (2), terapeuta ocupacional (1), odontólogo (1)
Cuidadoras/es	38	63 (38-88)	30	8	Madres (15), padres (5), parejas (6), tías (2), abuelas (2), suegra (1).
Familiares	16	44 (21-72)	15	1	Madres (4), padres (3), abuelas (3), abuelos (2), suegro/a (2), bisabuelas (2), pareja (1), vecina (1)

APS: atención primaria de salud.

### Técnicas de producción de datos

Entre agosto de 2021 y enero de 2023 se realizaron grupos focales por tipo de informantes, así como entrevistas semiestructuradas individuales, en forma presencial o en línea, según la factibilidad de aplicación de estas técnicas en el contexto de la pandemia COVID-19 en Chile (Tabla 2). Los grupos focales de los profesionales, todos realizados en forma en línea, se dividieron en dos grupos cuando la asistencia fue mayor a 9 personas, procurando que en cada grupo hubiera participantes de distintas profesiones. En el caso de los grupos focales de cuidadores y de familiares, la mayoría se realizaron de manera presencial, agrupándolos por comuna.

**Tabla 2:** Descripción de la muestra según Índice de Prioridad Social (IPS) y tipo de participante.

IPS	Profesionales y técnicos equipo de APS (n)	Cuidadoras/es (n)	Familiares (n)
Alta prioridad social: La Pintana y Cerro Navia	2 grupos focales (12)	2 grupos focales (12)	2 entrevistas individuales (2)
Media alta prioridad social: El Bosque y Recoleta	2 grupos focales (12)	2 grupos focales (15)	1 grupo focal (4)
Media baja prioridad social: La Granja y Renca	2 grupos focales (10)	1 entrevista indivdual (1)	1 entrevista individual y 1 grupo focal (3)
Baja o sin prioridad social: Macul y Providencia	2 grupos focales (11)	2 grupos focales (9)	1 grupo focal (5)

APS: atención primaria de salud.

La técnica de grupos focales permite incluir en un mismo momento a varios participantes que interactúan mostrando similitudes y diferencias en los contenidos que se abordan. Ante la imposibilidad de realizar entrevistas grupales, se realizaron individualmente utilizando el mismo guion temático flexible que integra temas comunes y preguntas específicas según tipo de participantes (Cuadro 1). Así, se facilitó la comparación entre grupos, identificando las particularidades de cada experiencia y apoyando con ello a la triangulación de datos.

**Cuadro 1:** Síntesis del guion de entrevistas con relación al cuidado de personas mayores en fin de vida y la preparación para la muerte.

DIMENSIÓN 1: CUIDADO DE PERSONAS MAYORES EN FIN DE VIDA			
Subdimensión	Temas iniciales		
	Equipos	Cuidadoras	Familiares
1.1. Prácticas de cuidado que realizan habitualmente	Actividades de asistencia que realizan con las personas mayores en fin de vida.	Relación que tenía con esa persona y las actividades realizaba en un día común.	Relación que tenía con esa persona y las actividades que realizaba habitualmente con él/ella.
1.2. Significados de los cuidados que realizan habitualmente	Propósito por el que realizan esas actividades.	Significado de haber cuidado a esa persona.	Significado del cuidado de una persona mayor en fin de vida.
1.3. Expectativas sobre los cuidados de personas mayores	Otras actividades que creen sería bueno realizar para asistir a personas mayores en fin de vida.	Apoyos que les hubiera gustado recibir para el cuidado que realizaron.	Apoyos les hubiera gustado que el/la cuidadora principal haya recibido. Apoyos que como familiar les hubiera gustado haber recibido.
DIMENSIÓN 2: PREPARACIÓN PARA LA MUERTE			
Subdimensión	Temas iniciales		
	Equipos	Cuidadoras	Familiares
2.1. Significados de la preparación para la muerte	Ideas o creencias sobre estar preparados para la muerte.	Creencias y sentimientos respecto de la posibilidad de prepararse para la muerte.	Creencias y sentimientos respecto de la posibilidad de prepararse para la muerte.
2.2. Prácticas de apoyo a la preparación para la muerte que realizan habitualmente	Actividades de apoyo y de preparación para la muerte que realizan con cuidadoras principales y con familiares de personas mayores en fin de vida.	Apoyo percibido desde el equipo de APS para prepararse para la muerte de la persona mayor que estuvo a su cuidado.	Apoyo percibido desde el equipo de APS para prepararse para la muerte del familiar mayor en fin de vida.
2.3. Expectativas/Recomendaciones sobre la preparación para la muerte	Otras actividades que creen sería bueno realizar para la preparación para la muerte de cuidadoras y familiares.	Necesidades y expectativas de apoyos para ellos/as y para la familia en relación con la preparación para la muerte. Recomendaciones para otras cuidadoras.	Necesidades y expectativas de apoyo para la familia en relación con la preparación para la muerte. Recomendaciones para otras familias.

APS: atención primaria de salud.

Las conversaciones fueron conducidas por un investigador(a) con formación y experiencia metodológica y acompañadas por otro(a) con experiencia clínica en salud mental, para pesquisar en los participantes alguna necesidad emergente de atención, consejería o derivación a propósito de la temática abordada. Todas las conversaciones fueron grabadas en audio.

El número final de grupos focales y entrevistas cumple con el criterio de saturación de la información por significados ^27^, discutido junto al análisis de datos en reuniones del equipo investigador.

### Análisis de los datos

A partir de la transcripción de las conversaciones, se realizó un análisis de contenido cualitativo de tipo semántico, aplicando un sistema de categorías y codificación construido, principalmente, de forma inductiva, que posteriormente fue sometido a la validación inter-codificadores. Con el objeto de triangular el análisis ^28^, los datos fueron codificados separadamente por al menos dos investigadores, luego fueron revisados en equipo.

Finalmente, se utilizó la estrategia de comprobación de participantes ^29^, entendida como la contrastación de los datos e interpretaciones con los sujetos, a través de un taller de devolución donde se presentaron los resultados y se facilitó un proceso de co-construcción ^30^ para evaluar el sentido y veracidad de las interpretaciones.

## Resultados

La preparación para la muerte fue considerada como una imposibilidad, centrándose en la incapacidad de evitar el sufrimiento que acompaña la muerte de un familiar. No obstante, al profundizar en las conversaciones, se reconocen algunas situaciones que ayudan a aceptar y esperar la muerte con tranquilidad, de las personas mayores y de sus familiares, especialmente del cuidador(a).

Las diferencias socioeconómicas de las comunas no parecen tener un rol diferenciador, especialmente en cuidadoras(es) y familiares. Los equipos de APS que contaban con mayores recursos para la atención domiciliaria, no necesariamente vinculado a comunas con más recursos económicos, presentaban mejores condiciones para la atención en general, no así para las prácticas de preparación para la muerte en específico.

Las experiencias relatadas se organizaron en tres categorías para profundizar en los significados y prácticas sobre la preparación para la muerte. Son ellas: (1) significados sobre la muerte e implicaciones para la preparación para la muerte; (2) prácticas de preparación para la muerte de las personas mayores en fin de vida; y (3) prácticas de acompañamiento a las cuidadoras y famílias.

### Significados sobre la muerte e implicaciones para la preparación para la muerte

La muerte es un tema difícil de tratar, que suele ser evitado. Su complejidad está asociada con sentimientos encontrados; el dolor de la pérdida y el fin del sufrimiento, que implican diferentes prácticas consideradas mediadoras de la oreparación para la muerte, aun cuando no son reconocidas como tales.

#### El dolor de la muerte

El dolor que implica la muerte restringe las posibilidades de preparación. Los equipos sanitarios señalan que las familias tendrían preconceptos de la muerte como tema tabú, del cual poco se habla. Asimismo, las cuidadoras(es) y familiares señalan que, aun sabiendo que la muerte es próxima, no pueden o quieren aceptarla por la intensidad del dolor de la pérdida, provocando que duden sobre la posibilidad de prepararse.

“*Aunque sabemos lo que va a pasar, pero a lo mejor no lo queremos aceptar, no queríamos aceptar que se muriera.* (...) *Yo no me puedo preparar… No queremos. No sé, no sé, en realidad*” (Grupo focal familiar, Renca).

En otros casos, rotundamente se rechaza esta posibilidad debido a la intensidad de la emoción y sentimientos que genera la pérdida.

“*Uno dice que se prepara, pero el momento que ya parten los viejitos uno siente el dolor, como hijo* (...) *le pueden aconsejar, hablar mil cosas. Pero en ese momento, en esa reacción, uno actúa como ser humano y siente el dolor*” (Grupo focal cuidador, El Bosque).

Existiría una diferencia entre tomar conciencia de la muerte y aceptarla sin sufrimiento. La experiencia dolorosa de la muerte de un ser querido restringe la comprensión de la preparación como una forma de no sufrir; en este sentido, nadie podría prepararse para la muerte. No obstante, muchos refieren sentirse preparados respecto a su propia muerte, lo que apoya la asociación de la preparación para la muerte de un otro con la idea de vivir el duelo sin la aflicción propia de este proceso.

Si bien todos los equipos de salud han tenido alguna experiencia con la muerte en sus labores, pocos han desarrollado estrategias concretas para abordarla y no han favorecido conversar sobre la muerte. Señalan actuar bajo sus propias creencias y sentido común, desconociendo la noción de preparación para la muerte y reconociendo la dificultad e importancia de hablar de la muerte.

“*Hablar de la muerte alivia, pero mi sensación es que es un pensamiento poco difundido, más bien nos negamos hablar de la muerte, pero cuando se habla lo agradecen. No se atreven, pero si uno da el primer paso después dicen “¡ay que bueno que puso este tema!*” (Grupo focal equipo APS, Macul).

#### Muerte como fin del sufrimiento

A pesar del dolor que puede generar perder a un familiar, las personas cuidadoras y sus familias definen la muerte como fin de un sufrimiento, lo que contribuye a aliviar la tristeza de la pérdida.

“*Uno dice ‘ya está descansando’, pero sigue triste, porque obvio, uno nunca está preparado para ese momento final*” (Grupo focal equipo de salud, La Pintana).

Para ellos, la condición de salud de la persona mayor -particularmente con una patología terminal, evidenciar el sufrimiento y los deseos de morir, les permitió asimilar la muerte como un evento esperado que traería descanso. Sin embargo, el alivio que podría implicar la muerte no necesariamente es asumida como “estar preparado”, sino más bien como una resignación.

“*Una nunca está preparada, pero de repente cuando llega ese momento tú dices ‘no, no es vida para ella, no es vida que esté así’*” (Entrevista con cuidadora, La Granja).

Más que una preparación, existe una resignación y consuelo de la muerte ante el sufrimiento de la agonía.

#### Creencias religiosas sobre la muerte

Para cuidadoras(es) y familiares, las creencias de tipo religiosas o espirituales contribuyen con un proceso de aceptación o alivio ante la muerte. Los equipos sanitarios enfatizan la importancia de la espiritualidad y de ajustarse a las creencias de las familias para facilitar la conversación sobre la muerte.

“*Pedía a Dios que se acordara de él, pedía a mis abuelos que ya lo tomaran de la mano, y le hablaba a mi papá, le decía “pero quédese tranquilo, mire, vaya* (...) *lo están esperando mis abuelos, mi mamá, mis tíos*” (Grupo focal cuidadora, Recoleta).

Las creencias religiosas sobre la muerte se asocian a una idea de continuidad hacia otro plano o mundo, implicando un encuentro con Dios y con familiares fallecidos que vendrían a buscar a quien está por morir. Además, sería un deseo que se solicita a un ser divino o familiares, invitando a la persona mayor a estar tranquila y partir donde otros le esperan. Estos significados aportan consuelo y muestran un anhelo, contrarrestando el aceptar la muerte y favoreciendo su preparación.

“*Es más fácil hablar con el cuidador o con el paciente si en la familia profesan alguna religión, no importa la religión, pero que tengan una fe* (...) *es más fácil decir que se aferren a la fe, que* [la muerte] *es un proceso que tenemos que vivirlo todos*” (Grupo focal equipo de salud, La Granja).

Las creencias religiosas de las familias facilitarían que el equipo de salud pueda abordar la muerte.

### Prácticas de preparación para la muerte de las personas mayores en fin de vida

Los equipos sanitarios destacan que las prácticas de preparación para la muerte dirigidas hacia las personas mayores son escasas, por falta de formación y por carecer de una definición programática. Las prácticas que emergen de las experiencias relatadas dan cuenta de iniciativas individuales y algunas consensuadas a nivel de equipo.

#### Silencio ante la muerte y tensión de la autonomía

Las prácticas dirigidas hacia las personas mayores dependen de su estado de salud, considerando la participación de sus familias.

“*Dependiendo del paciente, de cómo esté y de lo que quiera la familia también, se le explica todo lo que pueda pasar y las acciones que podemos hacer para mejorarlo entre comillas*” (Grupo focal equipo de salud, Providencia).

“*Se trata de conversar un poco con los familiares que es importante dar a conocer el diagnóstico* (...) *sobre todo si son usuarios que son autónomos y que pueden tomar la decisión* (...) *con pacientes que tienen demencia, por ejemplo, se hace un poquito más complejo*” (Grupo focal equipo de salud, Recoleta).

La información del pronóstico y de las atenciones sanitarias varía según las condiciones de salud y nivel de autonomía de la persona mayor, mostrando la complejidad que implica el manejo de información sensible por parte del equipo sanitario.

En pacientes sin compromiso de conciencia, se releva el derecho que tienen las personas mayores de conocer su diagnóstico y su pronóstico vital, aun cuando sea difícil de sobrellevar.

“*Le pregunto si quiere saber* (...) *Hay acciones que se hacen directamente con el paciente, porque ese paciente tiene que elegir si quiere morir de una sepsis en su cama o si quiere hacerse una limpieza quirúrgica, son escenarios distintos, los dos muy duros*” (Grupo focal equipo APS, Macul).

Para las cuidadoras(es) no es fácil abordar la muerte con las personas mayores; evitan mencionar o demostrar que la muerte está próxima, incluso reprimen la expresión de tristeza y buscan un entorno alegre, posiblemente como forma de ocultar esta realidad.

“[A su hermano] ‘*¿Cómo se te ocurre llorar delante de mi papá? Mi papá nos tiene que ver alegres’.* [Él respondía] *‘a mi papá no le queda mucho’. ‘Pero cállate’, le decía yo*” (Grupo focal cuidadora, La Pintana).

En otros casos se expresa lo contrario, incluso las propias personas mayores hablan de su muerte y sus deseos de morir.

“*Su papá la preparó, porque él le decía: ‘Si yo no estoy aquí, yo quiero que tú estés preparada para esto’. Él la preparó bien, hace ya harto tiempo atrás,* (...) *le dijo todo lo que tenía que hacer cuando él falleciera*” (Entrevista familiar, La Pintana).

“*Días antes ella me dijo que estaba cansada, que quería morir*” (Grupo focal cuidador, Macul).

Que las personas mayores puedan preparar a sus familiares para enfrentar la muerte, es coherente con la idea de estar preparados para su propia muerte y no la de otros. Quien va a morir lo acepta y conversa sobre ello, indicando a las demás personas qué hacer, qué esperan o desean.

#### Búsqueda de tranquilidad como medio para la preparación para la muerte

Existe acuerdo entre los participantes sobre que la preparación de la persona mayor para su propia muerte es un proceso que busca disfrutar los últimos momentos, en un entorno de afecto y tranquilidad.

“*En lo posible en paz, reconciliarse… Por ese motivo llamé a mi hermano, por último, para que ellos tuvieran tiempo… No sé si tenía que pedirle perdón, para que mi mamá pudiera partir*” (Grupo focal cuidadora, El Bosque).

“*Estar lo más en paz consigo mismo, o sea, qué temas hay que resolver o elegir alguno, porque quizás hay muchos, pero por lo menos tratar de sanar algunas cosas que están pendientes* (...) *es una forma de ir cerrando ciertos cabos que pueden generar esta diferencia al momento de irse*” (Grupo focal equipo de salud, Providencia).

Las prácticas para buscar paz o tranquilidad son la reconciliación, el perdón, resolver temas pendientes, y se incentivan por el equipo de salud y por las cuidadoras(es) bajo la creencia que facilita el paso a la muerte.

#### Preferencias fúnebres como medio para la preparación

Comúnmente las conversaciones de las personas mayores fueron sobre sus decisiones respecto a los servicios fúnebres y menos sobre sus deseos sobre los cuidados y la muerte.

Para las cuidadoras(es) y familiares, conocer estas preferencias ayuda a organizarse, anticipar algunas labores y aceptar la muerte. Abordar asuntos concretos, como el lugar o la ropa para el entierro, materializa la muerte como algo próximo.

“*Él estuvo plenamente de acuerdo en que yo comprara los funerales, siempre estuvimos de acuerdo*” (Grupo focal cuidadora, Providencia).

“*A mi papá le gustaban los trajes, nosotros lo teníamos preparado hace mucho rato. Con el dolor que significa planchar esa camisa que en algún momento se va a usar, pero al momento que llega uno estaba tranquila*” (Grupo focal cuidadora, Cerro Navia).

El equipo de salud contribuye con esta materialización por medio de la entrega de información sobre trámites básicos.

“*El mensaje es ‘tenga la cédula de identidad a mano, sepa dónde está’, cómo se hace el certificado de defunciones, el explicar que las funerarias se encargan prácticamente de todo, el conversar, si quieren cremarse o quieren ser enterrados*” (Grupo focal equipo de salud, Macul).

### Prácticas de acompañamiento a las cuidadoras y familias

El equipo sanitario reconoce una falta de recursos para apoyar integralmente a las personas mayores y sus familiares. Las cuidadoras(es) y familiares valoran el apoyo de la APS, a la vez que perciben la sobrecarga de los equipos y, en consecuencia, la falta de continuidad de la atención y seguimiento.

Los equipos sanitarios se enfocan principalmente en el cuidado de la persona mayor y su cuidador(a), a la vez que enfrentan desafíos para involucrar a todo el grupo familiar. Muchas cuidadoras(es) sienten que sus familias no les brindan suficiente apoyo, pero en contextos con mayores desafíos sociales, como la delincuencia o el abuso de drogas, la participación familiar se vuelve más complicada.

#### Acompañamiento psicológico y espiritual durante el cuidado

Las labores de cuidado contribuyen a la preparación para la muerte, pues se atestiguan los cambios que se producen en la persona mayor. Desde la perspectiva del equipo sanitario, quienes han cuidado con dedicación y afecto se sienten más preparados, dado que “hicieron todo lo posible” por su bienestar. Para las(os) cuidadoras(es), comprender el cuidado como “entrega total”, implica tranquilidad de haber hecho lo necesario para un buen vivir/morir, y sufrir menos, pues tienen satisfacción con su dedicación.

“*Me siento feliz de haber hecho lo que hice por mi mamá, me siento tranquila y sé que ahora descansa*” (Grupo focal cuidadora, El Bosque).

“*Uno sufre menos, porque queda con el corazón llenito de haber contribuido al proceso y al cuidado. Independiente del deterioro, de las sombras, de lo mal que lo pasa, de que pides SOS y no llega*” (Grupo focal cuidadora, Macul).

Estar satisfecho con los afectos y cuidados entregados tiene relación con “disfrutarlos en vida”, lo que involucra pasar tiempo con la persona mayor, hacerle cariño, entre otras expresiones afectivas de cercanía. Sin embargo, no implica que la labor no haya sido desgastante y muchas veces solitaria.

Los equipos también incentivan a las familias a ser parte de los cuidados, pues observan que les permitiría evidenciar las necesidades y de este modo prepararse.

“*Haber hecho todo lo que tenían que hacer* (...) *ser partícipe del proceso aportando, y el hecho de aportar los hace llegar al momento definitivo de mejor forma que las personas que miraron de lejos*” (Grupo focal equipo de salud, La Pintana).

Diferencian entre quienes observan el proceso y quienes aportan en los cuidados, lo que refuerza la idea de satisfacción y la necesidad de involucrar a las familias para afrontar la muerte, promover la corresponsabilidad de los cuidados y aliviar afectiva y laboralmente a quienes cuidan.

#### Cerrar los ciclos

Los equipos de salud consideran importante el acompañamiento después del fallecimiento, pero no siempre disponen de tiempo y recursos para ello. No obstante, se realizan prácticas personales o acordadas como equipo, como realizar una última visita domiciliaria, continuar el vínculo con cuidadoras en talleres o con apoyo psicológico. Familiares y cuidadoras(es) reconocen la necesidad de contar con apoyos psicológicos tanto antes como después de la muerte, aludiendo a la intensidad de los sentimientos de pérdida.

“*Nos preocupamos de cerrar con una visita* (...) *No siempre lo podemos hacer* (...) *no siempre se tienen los tiempos, en ninguna parte está considerado una visita de atención del duelo. Creo que es algo importante, porque uno genera un vínculo con los cuidadores a veces más que con el paciente*” (Grupo focal equipo de salud, Cerro Navia).

## Discusión

Este estudio buscó describir significados y prácticas sobre la preparación para la muerte de personas mayores en el contexto de la salud primaria. Los hallazgos resaltan la resistencia inicial hacia la idea de prepararse para la muerte; aunque se realizan acciones de preparación, estas no se perciben como tal. Los equipos sanitarios desconocen intervenciones de preparación para la muerte, mientras que las familias y cuidadoras(es) rechazan la idea de prepararse, temiendo que pueda disminuir el dolor de la pérdida.

Los significados sobre la muerte implican un desafío para la atención domiciliaria. Los equipos deben acompañar en el dolor que implica la muerte y en la aceptación del deterioro y los deseos de las personas mayores de morir, siendo la espiritualidad de las familias un recurso disponible. Es necesario contar con profesionales preparados para abordar la muerte desde una perspectiva integral, especialmente cuando comprenden la espiritualidad como fuera de sus límites de acción ^31^. En nuestro estudio, abordar la espiritualidad facilita hablar de la muerte, no obstante, lo habitual fue no hacerlo anticipadamente, lo mismo se observa a nivel hospitalario ^32^.

Las gradientes socioeconómicas intervienen en la severidad de los síntomas, en la carga de quienes cuidan y en los costes económicos ^18^. Sin embargo, la heterogeneidad socioeconómica de familias incluidas en este estudio no da cuenta de dichas diferencias en el cuidado y ni en la preparación para la muerte. Incluso las familias con mayores recursos viven la complejidad económica de los cuidados y la falta de corresponsabilidad.

Es importante cuestionar la pertinencia del concepto de preparación para la muerte, pues en este estudio se asocia con evitar el sufrimiento ante la pérdida de un ser querido. Evitando eufemismos, se podrían emplear otras formas de nombrar procesos de acompañamiento, familiarización y aproximación a la muerte en el diseño de programas, que incorporen un enfoque afectivo, que busque la tranquilidad sin pretender suprimir el dolor de la pérdida.

Más allá del nombre, el acompañamiento en esta etapa es necesario, con intervenciones psicoeducativas en los contextos de cuidados paliativos para aumentar habilidades, comunicación y apoyo emocional de cuidadores(as), además aliviar su carga y la calidad de la atención al final de la vida ^33^ ^,^ ^34^.

Este acompañamiento es un proceso, más que un estado final de preparación. Requiere tomar conciencia sobre la muerte inminente, no sólo desde aspectos considerados cognitivos, sino también afectivos y conductuales ^35^. En este estudio, el equipo de salud aborda la muerte desde sus propias intuiciones, enfatizando aspectos religiosos y, sobre todo, conductuales asociados a los procesos fúnebres. Parece ser que los aspectos afectivos son débiles o poco reconocidos como prácticas asociadas a un programa de acompañamiento domiciliario. En otros contextos, se ha asociado como barreras a la preparación: información poco clara, frustración con los servicios de cuidados paliativos y ambigüedad de la trayectoria de la enfermedad ^13^. En nuestro estudio, los equipos de APS señalan falta de directrices, tiempo y capacitación para incluir prácticas de acompañamiento de la muerte y duelo a personas mayores en fin de vida, cuidadores(as) y familiares.

Por otro lado, contar con profesionales que alertan a quien cuida sobre qué puede esperar de la muerte inminente, es considerado uno de los facilitadores de la preparación para la muerte ^13^. Según nuestros resultados, aún debemos avanzar en develar el tabú sobre la muerte, paso necesario para implementar programas de cuidados de fin de vida que incluya la definición de voluntades anticipadas. En esta tarea, la APS tiene una posición estratégica que podría favorecer la implementación de modelos de conversación de planificación anticipada a lo largo de la vida de una persona ^36^, contribuyendo a la preparación para la muerte en etapas anticipadas. La falta de capacidad del equipo de APS para abordar la preparación para la muerte, puede relacionarse con una carencia sobre la implementación de los cuidados paliativos. Si bien los cuidados paliativos y la APS deben reforzarse mutuamente, se debe tener en cuenta al menos tres ámbitos desafiantes: la preparación y formación en cuidados paliativos, y la colaboración entre profesionales; el desarrollo de habilidades para la comunicación y la delimitación de la relación con las familias; y la sobrecarga, falta de recursos y apoyos de los servicios de referencia ^37^.

En Chile los cuidados paliativos son relativamente recientes y, hasta hace poco, centrados en el nivel hospitalario asociado a cáncer terminal. El programa de apoyo a la dependencia de APS cumple labores de cuidados paliativos, pero no es su objetivo ^38^. Es necesario considerar los cuidados paliativos en la APS ^39^ y con ello incluir la preparación para la muerte. El año 2023 inició el Programa de Cuidados Paliativos Universales, enmarcado en la ley del mismo nombre (*Ley nº 21.375*) ^23^ que, si bien no contempla literalmente la preparación para la muerte, incluye el acompañamiento integral de la persona en fin de vida y su familia, específicamente en APS ^40^. Futuros estudios deberán indagar sobre su implementación, potencialidades y limitaciones.

Este estudio buscó una participación heterogénea y triangulación de actores, lo que favorece la comprensión profunda del fenómeno en estudio. No obstante, la distinción entre cuidadores y familiares evidenció una dificultad para convocar a aquellas personas menos involucradas. Los familiares participantes dan cuenta de la experiencia de quienes mostraron compromiso y responsabilidad con los cuidados, estando ausente la visión de familiares menos involucrados. Futuras investigaciones deberán profundizar significados y prácticas de las familias, con metodologías participativas o etnográficas, con miras a aportar a los programas de atención domiciliaria y la necesaria corresponsabilidad de los cuidados.

## Conclusiones

Este estudio permite concluir la importancia crítica de la preparación para la muerte en el contexto de cuidados domiciliarios. Hemos observado que, más allá de los aspectos médicos, la muerte en el domicilio requiere un acompañamiento afectivo y espiritualmente pertinente para propiciar un buen morir. Actualmente, Chile está implementando progresivamente la Ley de Cuidados Paliativos Universales (*Ley nº 21.375*) ^23^ y este estudio contribuye a relevar la necesidad de contar con recursos apropiados para que la APS lleve a cabo esta tarea con el objetivo de mejorar la calidad de vida y muerte de quienes enfrentan el final de sus días, brindando un apoyo efectivo y afectivo. En última instancia, señalamos la necesidad de repensar la formación de profesionales de la salud, destacando la importancia de dedicar más tiempo y recursos a la capacitación en el buen morir como parte integral del buen vivir.
